# Evaluation of a Direct, Rapid Immunohistochemical Test for Rabies Diagnosis

**DOI:** 10.3201/eid1202.050812

**Published:** 2006-02

**Authors:** Tiziana Lembo, Michael Niezgoda, Andrés Velasco-Villa, Sarah Cleaveland, Eblate Ernest, Charles E. Rupprecht

**Affiliations:** * University of Edinburgh, Midlothian, United Kingdom;; † Centers for Disease Control and Prevention, Atlanta, Georgia, USA;; ‡ Tanzania Wildlife Research Institute, Arusha, Tanzania

**Keywords:** rabies, diagnosis, immunohistochemistry, dispatch

## Abstract

A direct rapid immunohistochemical test (dRIT) was evaluated under field and laboratory conditions to detect rabies virus antigen in frozen and glycerol-preserved field brain samples from northwestern Tanzania. Compared to the direct fluorescent antibody test, the traditional standard in rabies diagnosis, the dRIT was 100% sensitive and specific.

In much of the developing world, rabies surveillance and diagnosis in domestic and wild animals are severely constrained. High ambient temperatures hinder the collection and preservation of fresh specimens. The use of the direct fluorescent-antibody assay (DFA), the traditional standard in rabies diagnosis (*1**,**2*), is limited by the costs of acquiring and maintaining a fluorescent microscope. Difficulties in obtaining diagnostic results from field material have led to widespread underreporting of disease.

Consequently, the true public health impact of rabies has been greatly underestimated (*3**–**5*), and political commitment for its control has been lacking. Moreover, the absence of a confirmatory test can result in the inappropriate management of animal bite injuries, with human deaths a potential consequence of delays in rabies postexposure prophylaxis (PEP) and unnecessary administration of PEP. The latter is a particular concern, given the scarcity and costs of human rabies vaccines and immunoglobulin in many parts of the world.

A rapid immunohistochemical test (RIT) to detect rabies virus (RABV) antigen has been developed in the Rabies Section of the Centers for Disease Control and Prevention (CDC) by incorporating various components of existing immunoperoxidase techniques (*6*). Like the DFA, the RIT is performed on brain touch impressions, but the product of the reaction can be observed by light microscopy, and RABV antigen appears as magenta inclusions against a blue neuronal background. The test recognizes all genotype 1 variants of RABV examined to date and all representative lyssaviruses. Modifications of a former indirect test have led to a direct test (dRIT) that uses a cocktail of highly concentrated and purified biotinylated anti-nucleocapsid monoclonal antibodies produced in vitro in a direct staining approach and allows a diagnosis to be made in <1 hour. For the routine diagnosis of rabies, glycerol saline is a convenient preservative in situations in which refrigeration or freezing facilities are not promptly available (*7*).

We report findings of a preliminary study to evaluate the dRIT, comparing results of the dRIT carried out under field conditions in Tanzania with the dRIT and DFA performed at CDC. The objectives were to validate the dRIT as a field test for rabies surveillance and evaluate the dRIT on glycerol-preserved field samples.

## The Study

Brain stem samples from various animal species were obtained from December 2002 to September 2004 as a result of rabies surveillance operations established in the Mara, Mwanza, and Shinyanga regions of northwestern Tanzania. Some archived glycerolated specimens were also analyzed. Samples were collected by inserting a drinking-straw through the occipital foramen, according to World Health Organization recommendations (*7*) or by opening the skull.

Some specimens were frozen (–20°C). Other samples inside straws were placed into a solution of phosphate-buffered 50% glycerol and stored either at +4°C or at –20°C or kept at room temperature (25°C ± 5°C) for up to 4 months before refrigeration or freezing.

Samples were allocated to 4 groups, according to the method of preservation and whether the samples were tested in the field and at the CDC laboratory or at CDC only (Table 1). Group A samples were kept in glycerol solution for <15 months and washed in phosphate-buffered saline (PBS) before testing by dRIT in the field. They were then stored at –20°C for <5 months and retransferred into fresh glycerol for shipment. At CDC, the samples were kept in glycerol for <2 months and rewashed in PBS before retesting by both dRIT and DFA or DFA only. Group B samples were stored frozen for <6 months, processed by dRIT in the field, and placed into glycerol solution for shipment to CDC, where they were stored for 2 months before being washed in PBS and retested. Group C samples were preserved in glycerol solution for <60 months, shipped, and processed at CDC by dRIT and DFA without previous testing in the field. These samples were washed in PBS just before testing. Group D samples were stored at –20°C in the field for 2 to 24 months, shipped frozen, and tested at CDC by dRIT and DFA.

**Table 1:** Methods of sample preservation and number of samples processed*

Preservation	No. washes in PBS	No. samples tested		
		dRIT field	dRIT CDC	DFA test CDC
Group A. glycerol saline/frozen/glycerol saline	2	44	39	44
Group B. frozen/glycerol saline	1	10	10	10
Group C. glycerol saline	1	0	89	89
Group D. frozen	0	0	16	16

*dRIT, direct rapid immunohistochemical test; PBS, phosphate-buffered saline; DFA, direct fluorescent-antibody assay; CDC, Centers for Disease Control and Prevention.

A qualitative assessment of the samples was made before testing. Five specimens at a time were stained by dRIT at ambient temperature as described below. Touch impressions were made on glass microscope slides as described (*8*). The slides were air-dried, fixed in 10% buffered formalin for 10 min, dip-rinsed in wash buffer PBS with 1% Tween 80 (TPBS), immersed in 3% hydrogen peroxide for 10 min, and dip-rinsed in fresh TPBS. After dipping, the excess buffer was shaken from the slides and blotted from the edges surrounding the impression. This treatment was repeated after each rinsing step. The slides were incubated in a humidity chamber (a cover on a moistened paper towel on an even surface) with the MAb cocktail for 10 min, dip-rinsed in TPBS, incubated with streptavidin-peroxidase complex (Kirkegaard and Perry Laboratories, Inc., Gaithersburg, MD, USA) for 10 minutes and dipped in TPBS. A 3-amino-9-ethylcarbazole (AEC) stock solution was prepared by dissolving one 20-mg tablet AEC (Sigma-Aldrich Corp., St. Louis, MO, USA) in 4 mL N,N-dimethylformamide (Fisher Scientific International, Inc., Pittsburgh, PA, USA) and stored at 4°C. A working dilution was prepared by adding 1 mL AEC stock solution to 14 mL 0.1 mol/L acetate buffer (Polyscientific, Bay Shore, NY, USA) and 0.15 mL 3% hydrogen peroxide. The slides were incubated with the AEC peroxidase substrate for 10 min and dip-rinsed in distilled water. They were then counterstained with Gill's formation #2 hematoxylin (Fisher Scientific International) diluted 1:2 with distilled water for 2 min and dip-rinsed in distilled water. Finally, they were mounted with a water-soluble mounting medium (BioMeda Corp., Foster City, CA, USA) and examined by light microscopy (Leica Microsystems AG, Wetzlar, Germany) in Tanzania and Axioplan 2 (Carl Zeiss AG, Göttingen, Germany) at CDC at magnifications of ×200 to ×400. The same operator performed the dRIT in the field and at CDC. However, at CDC, identification numbers unknown to the operator were assigned. The DFA (FITC Anti-Rabies Monoclonal Globulin, Fujerebio Diagnostic Inc., Malvern, PA, USA) was performed in a blind manner by another operator as described (*8*) and read by fluorescent microscopy (Axioplan 2).

Confidence intervals for the sensitivity and specificity were calculated by using the exact binomial distribution (S-Plus, Insightful Corp., Seattle, WA, USA). Of 159 total samples tested, 59 specimens (37.1%) were positive for RABV antigen, and 100 (62.9%) were negative by dRIT, with 100% agreement between the tests, whether dRIT was performed in field conditions only, both in field and laboratory conditions, or in laboratory conditions only. Assuming that the DFA was 100% sensitive and specific, the dRIT was 100% sensitive (95% confidence interval [CI] 93.9%–100.0%) and 100% specific (95% CI 96.3%–100.0%). Table 2 shows the distribution of positive samples in the various animal species.

**Table 2:** Number of Tanzanian brain samples processed by dRIT and DFA for different animal species*

Species	No. brains examined†
Domestic dog	73 (39)
Domestic cat	7 (3)
Cow	8 (7)
Goat	6 (5)
Livestock‡	1 (1)
Aardwolf (Proteles cristatus)	1
African civet (Civettictis civetta)	2
Banded mongoose (*Mungos mungo*)	2
Slender mongoose (Herpestes sanguineus)	3
Dwarf mongoose (*Helogale parvula*)	2
White-tailed mongoose (*Ichneumia albicauda*)	8 (1)
Mongoose‡	2
Black-backed jackal (*Canis mesomelas*)	3
Bat-eared fox (Otocyon megalotis)	8
Black-backed jackal/bat-eared fox‡	2 (1)
Cheetah (Acinonyx jubatus)	3
Small-spotted genet (*Genetta genetta*)	7 (1)
Lion (Panthera leo)	6
Serval (Felis serval)	1
Spotted hyena (*Crocuta crocuta*)	12 (1)
Striped hyena (*Hyaena hyaena*)	1
Zorilla (Ictonyx striatus)	1
Total domestic	95 (55)
Total wildlife	64 (4)
Total	159 (59)

*dRIT, direct immunohistochemical test; DFA, direct fluorescent-antibody assay. †The number of rabies-positive samples is shown in brackets. ‡Species not definitively identified.

**Figure 1:**
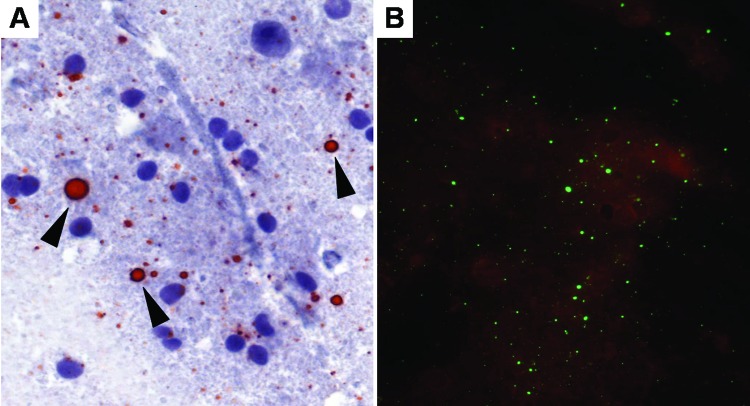
Touch impression of a rabies-positive Tanzanian domestic dog brain preserved in 50% glycerol saline solution for 15 months before testing by direct rapid immunohistochemical test (dRIT) and retested by direct fluorescent-antibody assay (DFA) after 5 months. A) Brain stained by dRIT: rabies virus antigen appears as magenta inclusions (arrowheads) against the blue neuronal hematoxylin counterstain. Magnification, ×630. B) Immunofluorescent apple-green viral inclusions in the same brain processed by DFA. Magnification, ×200.

The sensitivities of the dRIT and DFA were comparable, regardless of the method of preservation. We have no evidence that storage times affected positivity because 34 (77.2%) of 44 samples stored in glycerol solution remained positive for up to 10 months before being tested in the field and retested at CDC after an interval of up to 6 months. Furthermore, RABV antigen was successfully detected in the sample that had been preserved in glycerol for the longest duration (15 months) before dRIT in the field, stored frozen for 3 months before shipment to CDC, and kept in glycerol for 2 months before being retested (Figure 1). Similarly, viral inclusions were detected in a sample stored frozen for 24 months, although the antigen distribution was sparse with both tests. Our data do not provide any unequivocal conclusions on test sensitivity with samples preserved in glycerol solution for >15 months because results from all 15 archived brains were negative. For these samples, the presence of antigen at the time of collection cannot be excluded.

**Figure 2:**
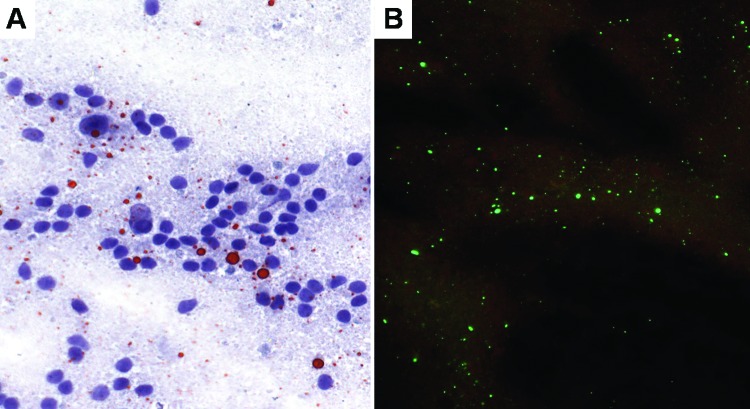
Touch impression of a deteriorated glycerolated brain from a Tanzanian spotted hyena (*Crocuta crocuta*) with rabies. A) Brain processed by direct rapid immunohistochemical test (dRIT). Magnification, ×400. B) DFA staining procedure on the same brain. Magnification, ×200.

Four of 10 (40.0%) deteriorated specimens were positive (Figure 2). Among the 6 brains with negative results, only 1 was suspected of containing rabies. The negative finding might have been caused by inadequate preservation, since the sample had been stored in glycerol solution at ambient temperature for up to 4 months before being refrigerated.

## Conclusions

The dRIT showed a sensitivity and specificity equivalent to those of the DFA. The test is simple, requires no specialized equipment or infrastructure, and can be successfully performed on samples preserved in glycerol solution for 15 months or frozen for 24 months and in variable conditions of preservation. These qualities make it ideal for testing under field conditions and in developing countries. Although further laboratory and field evaluations are required, our results are promising and highlight the potential value of the dRIT for countries with limited diagnostic resources. First, this technique could greatly enhance epidemiologic surveillance in remote areas where rabies incidence data are difficult to obtain. Second, the test could improve the ability to respond to outbreaks with effective management decisions. Third, it could be extremely valuable in guiding decisions regarding rational use of rabies PEP.
